# The Glasgow Royal Infirmary Competition

**Published:** 1901-02-23

**Authors:** 


### The Institutional Workshop.

### THE GLASGOW ROYAL INFIRMARY COMPETITION.

The troubles which have arisen in regard to the Glasgow Royal Infirmary Competition serve well to illustrate the difficulties in which hospital managers are apt to find themselves when confronted with the task of building or reconstruction. If they devote time and energy to the production of a very carefully drawn up and minutely complete code of instructions to the competing architects, these gentlemen find themselves tied down at every corner, the details having been already laid down in black and white, and feel that there is little left for them to do but to arrange an elevation. The result is that by dint of taking too much pains and laying down their conditions too strictly, the hospital managers deprive themselves of the very thing which ought to be the great advantage of a competition—viz. the wit and initiative of the architects themselves. "What can I do?" says the unhappy architect. "They tie me down as to the accommodation I am to provide, as to the size of the wards and rooms, as to the character of the ventilation, as to the mode of heating, and as to the disposition of the buildings on the land; and if I suggest a better way, or if I embody in my plans the results of my experience which teaches me that things could be done more conveniently on different lines, then my plan is disqualified!" Thus the careful committee fails to get the benefit of the knowledge of the architect, and, good as the building may look, it is, after all, an amateur effort. If, on the other hand, the committee arrange an open competition, stating only the amount of money which is to be expended and the amount of accommodation which they aim at, then they will get plenty of wit, an unbounded amount of initiative, and a heap of plans, some containing one good feature and some another; but probably no one plan fit to put up, for the chances are that the best and most experienced architects will not have entered the competition at all. If, again, they invite a few architects of known experience and position to send in competitive plans there is no doubt that they will get good plans. They know what they have to spend, they know the land, and they perhaps know how many patients they want to have. Knowing these things it seems a simple matter to put the whole affair into the hands of an experienced architect, and as the only way to satisfy the public that there is no favoritism is to have a competition, they think they had better ask a few good men to send in plans and then get some leading and generally trusted authority to decide which of the plans to adopt. By so doing they hope to clear themselves of all responsibility, which is all very well. But they must be prepared for this eventuality that they may find themselves tied down to a plan which, although fine enough from an architectural point of view, is anything but the sort of plan that they would desire or that the subscribers to the fund would care to see their money spent upon. The problem is thus by no means a simple one.

We can hardly doubt that much of the anger of the architects in regard to the Glasgow competition has arisen from the fact that after all the formalities of a competition, with an assessor and all the rest of it, had been gone through, the plan to which the assessor gave the first place was not accepted. The Glasgow Institute of Architects have issued a protest against the proceedings of the selection sub-committee, in which they draw attention to the fact that the competitors and the assessor were hampered in the exercise of their individual judgment by the conditions laid down, and that so faulty were these conditions that six out of the ten competing architects, in spite of the possible consequences—that is, in spite of the chance of being ruled out of the competition—found it necessary to entirely throw over the schemes furnished to them, and that among this number were all the four architects from outside Glasgow (two from London and two from Edinburgh), who were presumably invited specially on account of their knowledge of hospital design. But the architects especially protest against the setting aside by the sub-committee of the award given by the professional assessor.

This brings us round to the position of an assessor. As we have said, if a building committee wish to free themselves of responsibility they will call for plans from several architects and will give the final decision to an assessor. But if they do this they must be prepared to find themselves in the unfortunate position of having a plan palmed off upon them which does not meet their wishes; and this is just what happened at Glasgow for all the limitations they imposed. We do not express any opinion upon the Glasgow plans, but what we do insist upon is that the position which architects would like to see occupied by the professional assessor is a false one, and one which goes absolutely contrary to the old maxim that "he who pays the piper calls the tune." We do not think it possible for any committee to purge themselves of responsibility by throwing the choice upon an architect, however eminent. If a body of men collect money for a certain object their first duty is to see that that object is accomplished, and they do not perform this duty if they accept a plan which does not fulfil what they believe to be the main purposes for which the money has been subscribed. If, then, they embody these objects in their "conditions of competition" and these conditions are not complied with, they do nothing more than their duty in throwing out the offending plans. In fact, if the conditions are strictly drawn up, we think the assessor's duty should consist in seeing that in every detail these conditions are fulfilled and that no architectural mistake is committed rather than in deciding which plan is to be accepted. The final responsibility for this should, and, indeed, must always lie upon the committee. If the assessor picks out those plans in which the conditions are fulfilled, in which the architectural arrangements are such as will pass reasonable criticism and which can be erected for the price put upon them, he has done enough. Out of the plans so selected the committee must choose.

But if the committee do not draw up strict conditions; if they leave it to the competing architects to draw up the best plans they can for the money, still more must the responsibility of the final choice lie upon the committee; for under such circumstances the wildest plans may be sent in, and if they happen to tickle the fancy of the assessor they may be chosen. We have not specially investigated the plans of the Glasgow Royal Infirmary, but from what we have heard of them they contain points of which we should by no means approve. But in regard to the question whether the committee or the assessor is to decide we have no hesitation in saying that the Glasgow sub-committee is upholding a right principle in making its own selection. No committee can get rid of its responsibility by such a "confidence trick" as is involved in handing the decision of so important a matter over to an assessor, be he who he may. If the committee knows what it wants it must take care that it gets it. If, on the other hand, it has an open mind and waits to see what the competition will bring forth, its members are above all things bound, from the very multitude of alternatives offered them, to exercise their own judgment in making sure that they obtain what will best meet the wishes of the subscribers.

